# NR4A3 fusion proteins trigger an axon guidance switch that marks the difference between EWSR1 and TAF15 translocated extraskeletal myxoid chondrosarcomas

**DOI:** 10.1002/path.5284

**Published:** 2019-05-14

**Authors:** Monica Brenca, Silvia Stacchiotti, Kelly Fassetta, Marta Sbaraglia, Milijana Janjusevic, Dominga Racanelli, Maurizio Polano, Sabrina Rossi, Silvia Brich, Gian P Dagrada, Paola Collini, Chiara Colombo, Alessandro Gronchi, Annalisa Astolfi, Valentina Indio, Maria A Pantaleo, Piero Picci, Paolo G Casali, Angelo P Dei Tos, Silvana Pilotti, Roberta Maestro

**Affiliations:** ^1^ Unit of Oncogenetics and Functional Oncogenomics Centro di Riferimento Oncologico di Aviano (CRO) IRCCS, National Cancer Institute Aviano Italy; ^2^ Medical Oncology Department Fondazione IRCCS Istituto Nazionale dei Tumori Milano Italy; ^3^ Department of Pathology Treviso Regional Hospital Treviso Italy; ^4^ Unit of Experimental Molecular Pathology Fondazione IRCCS Istituto Nazionale dei Tumori Milano Italy; ^5^ Laboratory of Molecular Pathology Fondazione IRCCS Istituto Nazionale dei Tumori Milano Italy; ^6^ Department of Diagnostic Pathology and Laboratory Medicine Fondazione IRCCS Istituto Nazionale dei Tumori Milano Italy; ^7^ Department of Surgery Fondazione IRCCS Istituto Nazionale dei Tumori Milano Italy; ^8^ “Giorgio Prodi” Cancer Research Center University of Bologna Bologna Italy; ^9^ Laboratory of Experimental Oncology IRCCS, Istituto Ortopedico Rizzoli Bologna Italy; ^10^ Oncology and Haemato‐Oncology Department University of Milan Milano Italy; ^11^ Department of Medicine University of Padua School of Medicine Padova Italy

**Keywords:** extraskeletal myxoid chondrosarcomas, sarcoma, transcriptional profile, EWSR1, TAF15, NR4A3, axon guidance

## Abstract

Extraskeletal myxoid chondrosarcoma (EMC) is a rare sarcoma histotype with uncertain differentiation. EMC is hallmarked by the rearrangement of the *NR4A3* gene, which in most cases fuses with *EWSR1* or *TAF15*. *TAF15*‐translocated EMC seem to feature a more aggressive course compared to EWSR1‐positive EMCs, but whether the type of NR4A3 chimera impinges upon EMC biology is still largely undefined. To gain insights on this issue, a series of EMC samples (7 EWSR1‐NR4A3 and 5 TAF15‐NR4A3) were transcriptionally profiled. Our study unveiled that the two EMC variants display a distinct transcriptional profile and that the axon guidance pathway is a major discriminant. In particular, class 4–6 semaphorins and axonal guidance cues endowed with pro‐tumorigenic activity were more expressed in TAF15‐NR4A3 tumors; *vice versa*, class 3 semaphorins, considered to convey growth inhibitory signals, were more abundant in EWSR1‐NR4A3 EMC. Intriguingly, the dichotomy in axon guidance signaling observed in the two tumor variants was recapitulated in *in vitro* cell models engineered to ectopically express EWSR1‐NR4A3 or TAF15‐NR4A3. Moreover, TAF15‐NR4A3 cells displayed a more pronounced tumorigenic potential, as assessed by anchorage‐independent growth. Overall, our results indicate that the type of NR4A3 chimera dictates an axon guidance switch and impacts on tumor cell biology. These findings may provide a framework for interpretation of the different clinical–pathological features of the two EMC variants and lay down the bases for the development of novel patient stratification criteria and therapeutic approaches. © 2019 The Authors. *The Journal of Pathology* published by John Wiley & Sons Ltd on behalf of Pathological Society of Great Britain and Ireland.

## Introduction

Extraskeletal myxoid chondrosarcoma (EMC) is a rare sarcoma histotype (<3% of soft tissue sarcomas) that primarily occurs in the extremities of adults 1. EMC is defined as ‘an indolent, but resilient and capricious tumor’ 2, with a propensity to relapse even after several years from diagnosis and up to 40% risk of metastases 3, 4. Surgery is the mainstay treatment for primary localized EMC, while advanced disease requires medical therapy. Unfortunately, chemotherapy has a limited efficacy over time 4, 5 but promising results have been recently achieved with antiangiogenetics. Specifically, we recently reported on the long lasting activity of the tyrosine kinase inhibitor sunitinib in a retrospective series of 10 advanced EMC 3, and a European phase 2 study is investigating the activity of pazopanib (NCT02066285).

Originally considered a cartilaginous neoplasm 1, 6, EMC is currently classified as a tumor of uncertain differentiation 1. Although the histogenesis of EMC remains unclear, different reports have highlighted the presence of neural‐neuroendocrine features as evidenced by occasional staining for neuron‐specific enolase, chromogranin, synaptophysin and/or identification of dense‐core granules on ultrastructural analysis 7, 8, 9, 10. Neural‐neouroendocrine features were reported as enriched in EMC also in a microarray study comparing this tumor to other soft tissue sarcomas 11. Most EMC are hypocellular and classified as low‐grade neoplasms. However, high‐grade EMC are also described and characterized by hypercellularity and occasional rhabdoid morphology that correlates with adverse clinical outcomes 1, 12.

A distinctive feature of EMC is chromosome rearrangement involving the 9q22 region harboring the *NR4A3* gene. In most cases, the whole NR4A3 coding region is fused downstream of the N‐terminal transactivation domain of EWSR1, less frequently to the same domain of TAF15. Occasional fusions with *TCF12*, *TFG*, *HSPA8* have also been reported 1, 13, 14.

NR4A3 is a poorly characterized protein that, together with NR4A1 and 2, constitutes the NR4A family of orphan nuclear hormone receptors. Although there is a putative ligand binding domain, no endogenous ligand has been identified. NR4A proteins are involved in the control of different biological processes such as cell proliferation, migration, apoptosis, neuron development, axonogenesis and angiogenesis, and appear as emerging players in the context of cancer 15, 16. NR4A proteins, whose activities are context and tissue specific, localize both in the nucleus and in the cytoplasm. In the nucleus, they are reported to act as a transcription factors via binding to NBRE (NGF‐induced B factor‐response element) and related consensus sequences on DNA; in the cytoplasm, they have been shown to intersect different molecular pathways by protein–protein interactions, and increased cytosolic NR4A1 or NR4A2 protein levels have been associated with tumor aggressiveness 16, 17, 18. As EMC chimeras retain the DNA binding domain of NR4A3, they potentially recognize NBRE consensus sites 19, 20. EWSR1 and TAF15 are members of the FET family of RNA binding proteins that participate, with their N‐terminus, to the generation of a number of fusion oncoproteins involved in sarcomas 21.

Recent reports suggest that TAF15‐translocated EMC feature a more aggressive behavior compared to the EWSR1‐translocated counterpart 12. However, whether and how the type of NR4A3 chimera affects the tumoral phenotype is still largely undefined. To shed light on this issue and ideally provide grounds for better risk classification criteria and targeted therapeutic approaches, we molecularly profiled a set of EMC samples and cell models expressing either the EWSR1 or the TAF15 fusion transcript.

## Materials and methods

### Tumor series

The study was conducted on a series of 12 EMC retrieved from the pathology files of Fondazione IRCCS Istituto Nazionale dei Tumori (Milano), the Treviso Regional Hospital and the IRCCS Istituto Ortopedico Rizzoli (Bologna) and approved by the appropriate Institutional Review Boards. Clinicopathological features of the series are summarized in Table 1. All patients were treated with surgery for disease originating from soft tissues. Pathological diagnosis was centrally reviewed by two expert pathologists (APDT and SP) and the rearrangement of *NR4A3* was confirmed by FISH.

**Table 1:** EMC clinicopathological features

CASE #	NR4A3 partner	Gender	Age at the diagnosis	Tumor site
1	EWSR1	F	48	Upper leg
2	EWSR1	M	48	Groin
3	EWSR1	M	55	Upper leg
4	EWSR1	M	57	Upper leg
5	EWSR1	F	60	Upper leg
6	EWSR1	M	71	Buttock
7	EWSR1	M	76	Upper leg
8	TAF15	M	50	Buttock
9	TAF15	M	62	Lower leg
10	TAF15	M	58	Buttock
11	TAF15	M	39	Lower leg
12	TAF15	M	50	Lower leg

### Immunohistochemistry and FISH analyses

Representative 2‐μm sections of formalin‐fixed, paraffin‐embedded (FFPE) surgical samples were immunostained for Semaphorin 4D, Plexin A4, Synaptophysin, Reelin, Nestin, NCAM1/CD56 and Glial Fibrillary Acidic Protein as detailed in supplementary material, Supplementary materials and methods. FISH analyses were performed on FFPE sections using the probes indicated in supplementary material, Supplementary materials and methods. At least 50 non‐overlapping nuclei were scored at ×100 magnification.

### Whole transcriptome sequencing and gene functional annotation

Tumor sections with >70% tumor nuclei were used for transcriptional analysis. Total RNA was isolated from FFPE EMC as in 22 and from frozen samples as in 3. RNA from cell cultures was extracted using the TRIzol reagent (ThermoFisher Scientific, Waltham, MA, USA). RNA‐sequencing (RNA‐Seq) was used for transcriptional profiling. For FFPE samples and cell cultures RNA‐Seq libraries were prepared as in 22 and sequenced on a Hiseq1000 Illumina apparatus to an average of 70‐million paired‐end reads per sample. Raw sequence data quality was assessed using the FastQC software (http://www.bioinformatics.babraham.ac.uk/projects/fastqc/). STAR, HTSeq, and DEseq2 were used for read mapping, quantification, gene‐level exploratory, and differential expression analysis 23, 24, 25. Raw and processed sequencing data are available at http://opendocuments.cro.it/cod/handle/item/9167.

DESeq2 was used for principal component analysis (PCA). Biomedical Genomics Workbench (QIAGEN‐Bioinformatics‐v4.1.1, Qiagen, Hilden, Germany) was used for additional quantifications (transcripts per million, TPM) and hierarchical clustering. For fresh‐frozen samples libraries were prepared and analyzed as in 3. Over‐representation analyses (ORA) were performed with DAVID (v6.7) 26 and WebGestalt‐2017 (ORA‐WebGestalt) 27. Gene set enrichment analyses (GSEA) were performed with WebGestalt‐2017 (GSEA‐WebGestalt) 27 and GSEA‐Broad Institute (v3.0) 28. Ingenuity pathway analysis (IPA) (QIAGEN) 29 and NetworkAnalyst 30 were used for further functional annotations. Details are provided in supplementary material, Supplementary materials and methods.

### Cells and constructs

tBJ/ER were maintained and engineered by retroviral infections as described 31, 32. The following cDNAs, cloned into the retroviral PLPCX vector (Clontech Takara Bio Inc., Kusatsu, Japan), were used: full‐length *NR4A3*; E‐N, corresponding to *EWSR1* (exons 1–12)‐*NR4A3* (exons 3–8); T‐N, corresponding to *TAF15* (exons 1–6)‐*NR4A3* (intron 2–exon 8) and T‐N*, corresponding to the commonest *TAF15* (exons 1–6)‐*NR4A3* (exons 3–8) fusion. Both T‐N and T‐N* encode the whole coding sequence of *NR4A3* (exons 3–8); T‐N retains a short cryptic exon located in *NR4A3* intron 2 (ENST00000395097.6 isoform), thus encoding 25 additional amino acids prior to the *NR4A3* ATG. Both untagged and Strep‐tagged versions of these plasmids were used.

For transcriptional profiling 4 (E‐N and T‐N) or 3 (NR4A3) biological replicates were generated by separate viral infections. Anchorage‐independent growth assay was carried out in soft agar‐semisolid medium as previously described 31. Colonies were scored at ×100 magnification 8 days after plating and size and number of colonies/field (cutoff size > 30 μm) were estimated. A minimum of 20 non‐overlapping fields of three independent replicates were scrutinized blindly by two investigators.

### Protein analysis

For western blot analysis, protein lysates were generated and separated on SDS‐PAGE as detailed in supplementary material, Supplementary materials and methods. Membranes were probed with the following antibodies: anti‐NR4A3 Mouse MoAb clone H7833 (R&D Systems, Minneapolis, MN, USA), Mouse MoAb clone OTI5C2 (Origene, Rockville, MD, USA) targeting the N‐terminus and C‐terminus of NR4A3, respectively; anti‐Strep‐Tag mouse MoAb (clone GT661, AbCam, Cambridge, UK); anti‐POLR3A MoAb (Rabbit MoAb, clone D5Y2D, Cell Signaling Technology, Danvers, MA, USA) was used to normalize total protein load.

### RT‐qPCR and transcriptional array analyses

Relative mRNA levels of *NR4A3*, related fusions and of a set of *SEMAs* were assessed by RT‐qPCR in tBJ/ER cell models. At least three independent biological replicates were analyzed. A targeted transcriptional array analysis was performed on 6 EMC for which suitable material was available. Methodological details on these procedures are in supplementary material, Supplementary materials and methods.

### Chromatin affinity purification‐quantitative PCR (ChAP‐qPCR)

The MatInspector software was employed to identify putative NR4A3 consensus sites (NBRE) 33. Chromatin affinity purification (ChAP) on tBJ/ER cells expressing Strep‐tagged NR4A3, EWSR1‐NR4A3 or TAF15‐NR4A3 was performed as detailed in supplementary material, Supplementary materials and methods. Precipitated DNA was quantified by qPCR with primers targeting the identified *SEMA3C* regulatory region. The fraction of the target DNA recovered from the input was measured by comparing the threshold cycle (CT) of the precipitated sample to a dilution of its own input, and was expressed as relative enrichment. The background was estimated by PCR amplification of an unrelated genomic region (*GAPDH* exon 1).

## Results

### EWSR1 and TAF15‐rearranged EMC feature a different gene expression pattern

**Figure 1:**
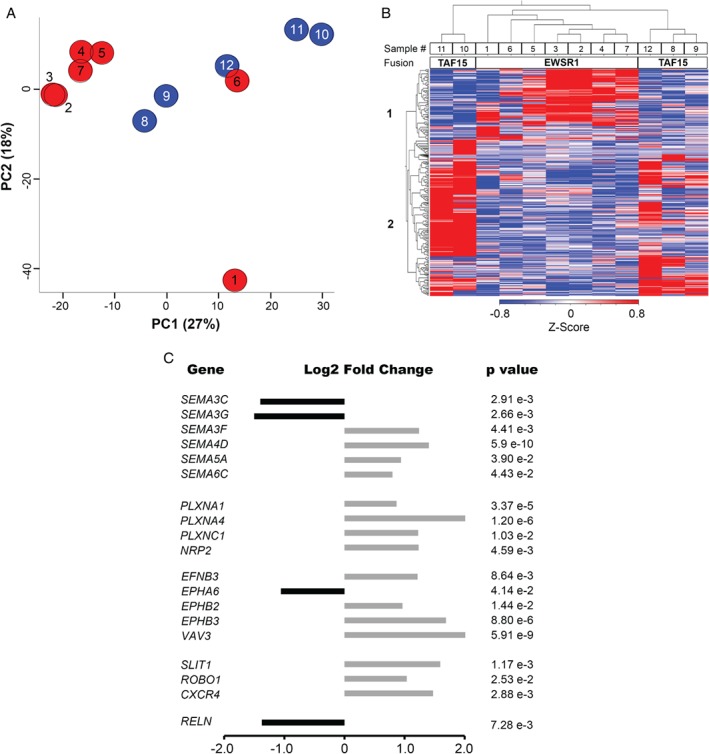
Transcriptome profiling of EMC. (A) Principal component analysis of the transcriptome of EWSR1‐NR4A3 (red) and TAF15‐NR4A3 (blue) rearranged EMC. (B) *Z*‐Score normalized heat map of the expression values (log2 transformed) of the top 500 differentially expressed genes in TAF15‐NR4A3 and EWSR1‐NR4A3 EMC. Sample (top) and gene (left) dendrograms are shown. Sample ID number and NR4A3 fusion partner are indicated. The color bar indicates the *Z*‐score and reflects the relative gene expression level, from blue (low), white (medium) to red (high). Gene cluster 1 consists of the genes overexpressed in EWSR1‐NR4A3 EMC; gene cluster 2, the genes overexpressed in TAF15‐NR4A3 EMC. (C) Log2 fold‐change of the axon guidance molecules differentially expressed in the two EMC variants. Black and grey bars indicate genes underexpressed and overexpressed, respectively, in TAF15‐NR4A3 versus EWSR1‐NR4A3 tumors.

To gain insight into EMC pathobiology, seven EWSR1‐NR4A3 and five TAF15‐NR4A3 EMC were transcriptionally profiled by RNA‐Seq (Table 1). For five samples (four EWSR1 and one TAF15) frozen material was also profiled, yielding similar results to the matched FFPE counterpart (data not shown). PCA of the transcriptome showed that, although there was not a net demarcation between TAF15 and EWSR1 EMC, the majority of EWSR1 tumors (5/7) tended to separate from the TAF15 EMC group along the Principal Component 1 (Figure 1A). Hierarchical clustering of the top 500 genes with differential expression between TAF15 and EWSR1 EMC (false discovery rate [FDR] < 0.1) provided a preliminary overview of the transcriptome pattern (Figure 1B). This scrutiny identified two major gene clusters: cluster 1 included 156 genes overexpressed in EWSR1 EMC; cluster 2 consisted of 344 genes expressed at higher levels in TAF15 EMC. Beside ‘generic’ biological processes (e.g. multicellular organism development, anatomical structure development), several of the top gene ontology (GO) terms of both clusters were related to neurogenesis; gene cluster 1, associated with EWSR1 EMC, also included muscle and circulatory system development (DAVID Gene Functional Annotation Tool, see supplementary material, Table S1A).

To gain a better insight, we then focused on genes that, besides being statistically differentially expressed in TAF15 versus EWSR1 tumors, had an absolute variation greater than 50% (abs.log_2_ FC > 0.6) (see supplementary material, Table S1B). Overall, functional annotation with different algorithms highlighted an enrichment of GO categories associated with axon guidance, neurogenesis, blood vessel morphogenesis and muscle system process (Table 2). In particular, over‐representation analyses (ORA‐WebGestalt and DAVID) indicated that nervous system development, axon guidance, Semaphorin‐Plexin signaling pathway, blood vessel morphogenesis and glutamate receptor signaling were among the top enriched GO biological processes (Table 2). Axonal guidance, which includes various components of VEGFR signaling, was the top enriched canonical pathway according to IPA. Other enriched pathways included Reelin, Ephrin and glutamate receptor signaling (Table 2 and supplementary material, Table S1C–E).

**Table 2:** Representative functional categories of the genes differentially expressed in TAF15‐NR4A3 versus EWSR1‐NR4A3 EMC and in T‐N versus E‐N cell models

Sample	Method	Tool	GO biological process/pathway	FDR
EMC	ORA	WebGestalt	Neuron projection guidance	1.3e−3
			Semaphorin‐Plexin signaling	1.6e−3
			Axon development	1.8e−3
			Blood vessel morphogenesis	1.5e−2
		DAVID	Neuron system development	1.4e−10
			Neurogenesis	4.4e−3
			Neuron differentiation	1.8e−2
			Neuron projection	3.9e−1
	GSEA	WebGestalt	Muscle system process	1.1e−3
			Axon development	1.7e−2
			Neuron projection guidance	2.3e−2
			Nerve development	2.0e−1
		GSEA‐Broad	Neuron projection morphogenesis	4.5e−3
			Axon	4.0e−2
			Extracellular space	1.8e−1
			Muscle system process	1.6e−1
	IPA	IPA Canonical pathways	Axonal guidance signaling	1.1e−5
			Glutamate Receptor Signaling	2.0e−4
			Reelin signling in neurons	2.0e−3
			Ephrin receptor signaling	3.6e−3

Cell models	ORA	WebGestalt	Cell growth	2.0e−5
			Neural tube development	4.1e−5
			Blood vessel morphogenesis	5.1e−5
		DAVID	Neurogenesis	1.1e−5
			Axon guidance	3.0e−3
			Blood vessel development	8.7e−3
	GSEA	WebGestalt	Blood vessel morphogenesis	1.0e−3
			Nerve development	1.4e−1
			Axon development	2.4e−1
		GSEA‐Broad	Angiogenesis	1.8e−2
			Nerve development	9.0e−2
			Neuron differentiation	1.3e−1
	IPA	IPA Canonical pathways	Axonal guidance signaling	5.8e−9
			ERK/MAPK signaling	1.8e−4
			VEGF ligand‐receptor interaction	1.9e−3

GSEA predicted that neuron development and axonogenesis were among the categories enriched in TAF15‐positive tumors, whereas muscle system and extracellular space (that includes several secreted factors involved in angiogenesis) were among the processes enriched in EWSR1 EMC (Table 2 and supplementary material, Table S1F–G).

**Figure 2:**
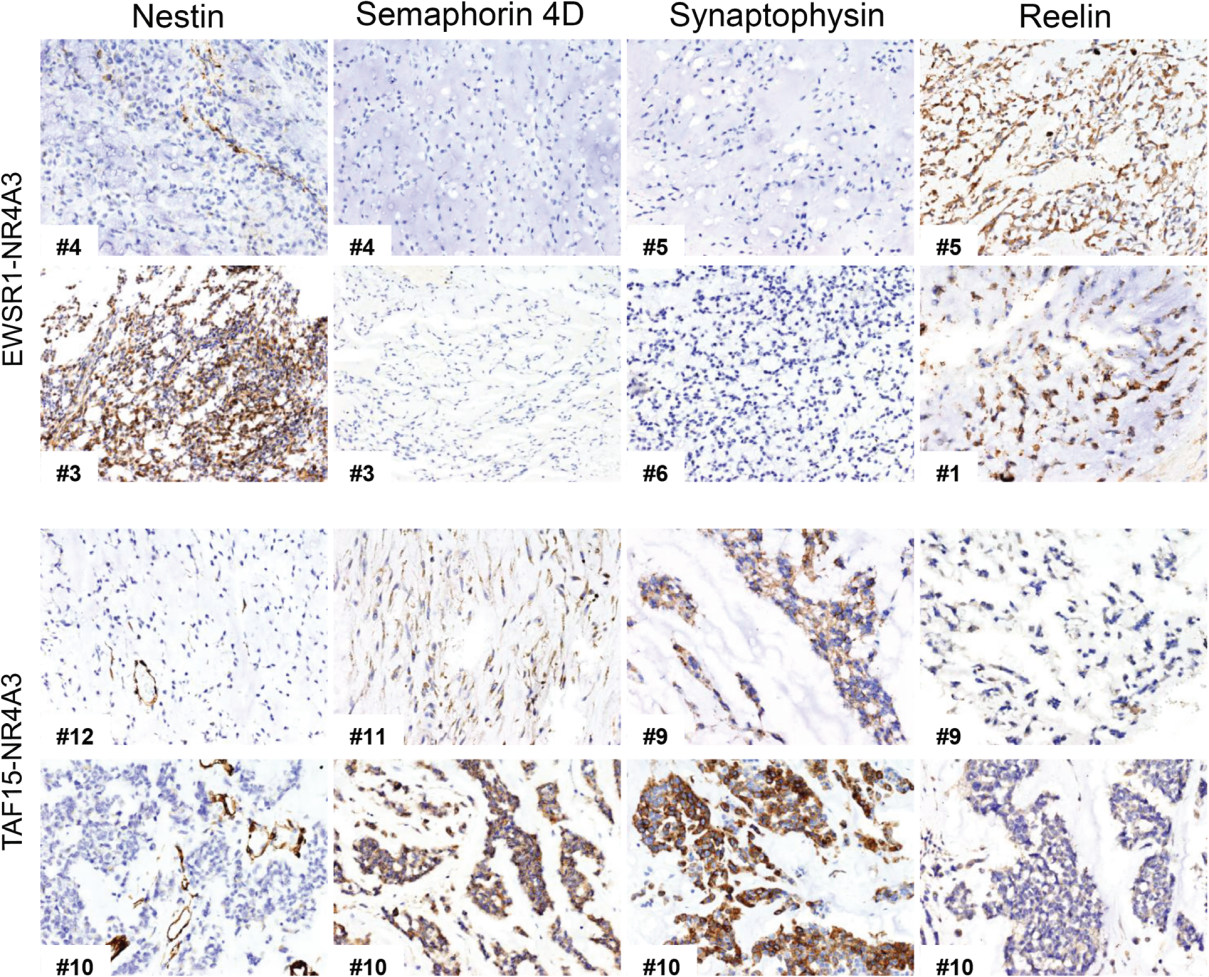
Representative EMC immunostainings representative immunostainings for Nestin, Semaphorin 4D, Synaptophysin and Reelin in a set of EWSR1‐NR4A3 (top) and TAF15‐NR4A3 (bottom) EMC. Case ID number is indicated. Magnification: ×100.

Axon guidance genes differentially expressed in the two EMC variants included Reelin (*RELN*), components of the Eph/Ephrin signaling network, semaphorins (*SEMA*) and cognate receptors and co‐receptors Plexin (*PLXN*) and Neuropilin (*NRP*), as well as other genes involved in the regulation of the vasculature. In particular, TAF15 EMC overexpressed class 4–6 *SEMA* as well as *PLXNA1, PLXNA4, PLXNC1, SLIT1*, and *ROBO1*. TAF15‐positive EMC also overexpressed a number of other neuron‐associated molecules such as *SYP, ELAVL2, NPDC1, SCG3, NEFH, STX1A*, and *UNC13A*. Conversely, *SEMA3C, SEMA3G*, and *RELN* were more expressed in EWSR1 tumors (Figure 1C; see supplementary material, Table S1H). This differential expression trend was corroborated by a targeted transcriptional array analysis on a subset of cases for which suitable material was available (data not shown). Immunohistochemical analyses (Figure 2 and Table 3) indicated an overall positivity of EMC for neuronal markers (Nestin, CD56/NCAM and GFAP) and confirmed that SEMA4D, Plexin A4, Synaptophysin (SYP) were higher in TAF15 compared to EWSR1‐translocated EMC. Conversely, the highest levels of Reelin were observed among EWSR1 positive tumors.

**Table 3:** EMC immunoprofile

Case #	NR4A3 partner	SEMA 4D	Plexin A4	Synaptophysin	Reelin	Nestin	CD56	GFAP
**1**	EWSR1	−	+	−	++	+	+++	−
**2**	EWSR1	−	+	+/F	++	+	+	−
**3**	EWSR1	−	+	−	+/F	++	−	+/F
**4**	EWSR1	−	+	−	+	+/−	+	+
**5**	EWSR1	−	+/−	−	+	+	−	+
**6**	EWSR1	−	+	−	+/−	+/F	−	−
**7**	EWSR1	−	+	+/F	+/F	+	+/F	+
**8**	TAF15	+	+	+/F	+/F	+	−	+/F
**9**	TAF15	+/F	+++	+	+/F	−	+	−
**10**	TAF15	++	+++	++	+	+/−	+	+
**11**	TAF15	+/F	++	+/−	+	+/−	−	−
**12**	TAF15	+	+	++	+	+/−	+	+/−

−, very weak to negative; +/−, weak; +/F, focal positivity; +, mild; ++, moderate; +++, strong.

### The type of chimera dictates biology and transcriptional profile of the two EMC variants

Based on these results, we hypothesized that the type of chimera dictates the different phenotype of the two EMC variants. To verify this hypothesis, in the absence of EMC‐derived cell cultures, we sought to engineer sarcoma cell lines for the expression of EWSR1‐NR4A3 and TAF15‐NR4A3. Unfortunately, while NR4A3 was well tolerated, most of the cell lines tested (U2‐OS, MES‐SA, VA‐ES‐BJ, HOS, HT‐1080) were refractory to the expression of NR4A3 chimeras (data not shown). Eventually, tBJ/ER transformed human fibroblasts, which are an effective model for studying mesenchymal cell transformation 31, turned out to be a suitable background. In these cells, ectopic NR4A3 and relative fusion proteins were expressed both in the nucleus and in the cytoplasm, with a prevalent nuclear localization (not shown).

**Figure 3:**
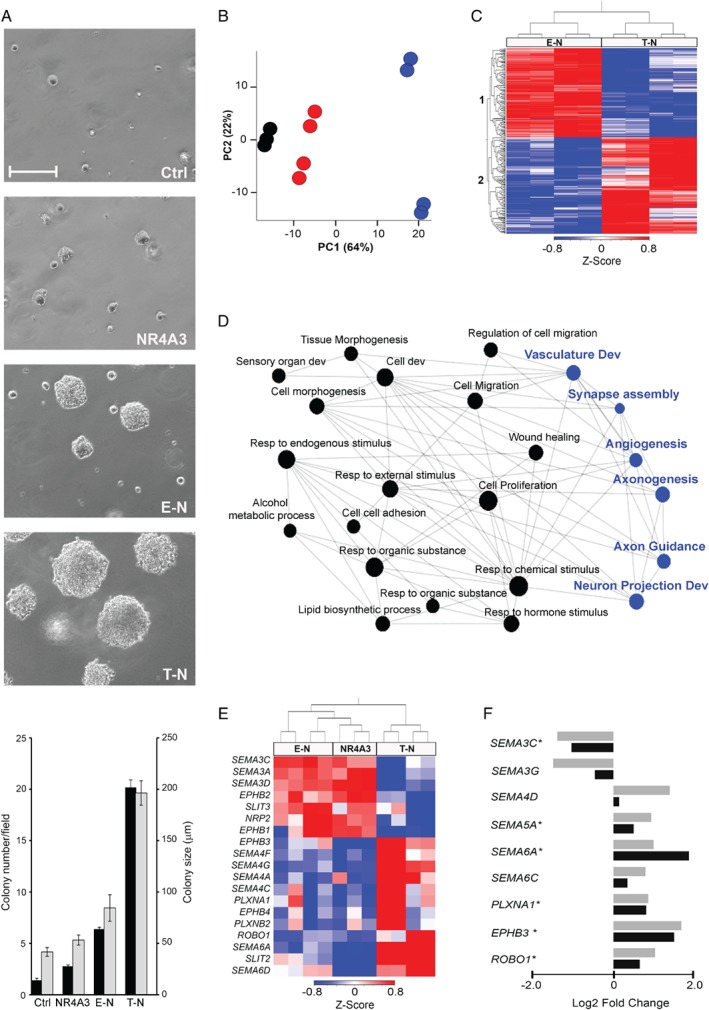
Transcriptome profiling of E‐N, T‐N and NR4A3 cell models. (A) Top: Representative phase‐contrast images showing colony formation in soft agar of tBJ/ER cells engineered to express control empty vector (Ctrl), NR4A3, E‐N or T‐N. Magnification: ×100; scale bar = 200 μm. The plot (bottom) shows mean number of colonies >30 μm ± SE (black bars) and mean colony size ± SE (gray bars) per ×100 magnification field at day 8 post‐plating. (B) PCA of the transcriptome of NR4A3 (black), E‐N (red) and T‐N (blue) cell models. (C) *Z*‐score normalized heat map of the top 500 differentially expressed genes in T‐N versus E‐N cells. Sample (top) and gene (left) dendrograms are shown. Color‐coding is as in Figure 1. Gene cluster 1 consists of the genes overexpressed in E‐N; gene cluster 2, genes overexpressed in T‐N. (D) Network integration analysis of genes differentially expressed in both EMC (TAF15‐NR4A3 versus EWSR1‐NR4A3) and in cell models (T‐N versus E‐N). Axon guidance‐associated GO biological processes are highlighted in blue. (E) Heat map and hierarchical clustering for the axon guidance molecules that are differentially expressed in T‐N versus E‐N. NR4A3 co‐clusters with E‐N. (F) Plot showing the modulation in tBJ/ER T‐N versus E‐N cells (black bars) of the axon guidance cues that were detected as statistically differentially expressed in TAF15‐NR4A3 versus EWSR1‐NR4A3 EMC (grey bars). *Genes whose modulation (Log2 fold‐change) is statistically significant also in cell models (*p* < 0.05).

The cell models generated in the tBJ/ER background, namely EWSR1‐NR4A3 (E‐N), TAF15‐NR4A3 (T‐N), and NR4A3, were tested for their tumorigenic potential by anchorage‐independent growth in soft agar. The expression of either chimeric protein conveyed an advantage in terms of colony formation efficiency as opposed to NR4A3 (Figure 3A). Moreover, the contrast T‐N versus E‐N indicated that cells expressing TAF15‐NR4A3 formed a greater number and larger colonies. These results were confirmed in independent biological replicates with untagged and Strep‐tagged versions of the constructs.

Given the more malignant phenotype of T‐N cells, we sought to compare the biological behavior of the two major TAF15‐NR4A3 isoforms detected in human tumors. Cells were engineered with the T‐N* fusion variant (*TAF15* exon 6‐*NR4A3* exon 3) and with the less common T‐N variant (*TAF15* exon 6‐*NR4A3* intron 2). Both T‐N and T‐N* were well expressed at mRNA and protein level (T‐N* even a little more than T‐N). T‐N and T‐N* were then compared for colony formation efficiency. Under these experimental conditions, T‐N* and T‐N were essentially indistinguishable (see supplementary material, Figure S1).

Transcriptome analysis of the tBJ/ER cell models revealed a distinct gene expression pattern. In particular, E‐N mapped close to NR4A3 whilst T‐N cells clustered apart according to PCA (Figure 3B). Hierarchical clustering of the top differentially expressed genes between E‐N and T‐N (top 500; FDR < 0.1) yielded two major clusters: cluster 1, composed of genes (240) overexpressed in E‐N and primarily associated with cell proliferation and vasculature development; cluster 2, made of genes up‐regulated in T‐N (260) and including molecules implicated in nervous system development (Figure 3C; supplementary material, Table S2A). Axonogenesis, nervous system and vasculature development were also called when all the genes differentially expressed in the two cell models (abs.log_2_ FC > 0.6; *p* < 0.05) were functionally annotated (Table 2 and supplementary material, Table S2B–G).

To address how well these models recapitulated human EMC, the list of the top significantly differentially expressed genes in T‐N versus E‐N cells and in the two tumor variants were intersected, revealing greater than 10% overlap (170/1500 genes). Not surprisingly, the shared genes turned out to be implicated in axon guidance, neurogenesis and angiogenesis (Figure 3D and supplementary material, Table S2H), strongly supporting a key role of these pathways as discriminant factors between EWSR1‐NR4A3 and TAF15‐NR4A3 positive contexts.

Several axon guidance factors such as *SEMAs*, *PLXNs* and *EPH/EFN* signaling molecules were modulated in response to the two chimeric genes. Although the molecules that were differentially expressed in the cell models were not precisely the same as in EMC, likely also because of the different cellular background, there was a remarkable overlap in the modulation of the classes of axon guidance cues. Specifically, as in tumors, class 4–6 *SEMAs* and class 3 *SEMAs* were in general more abundant in T‐N and E‐N cells, respectively, with an apparent trend of incremental divergence from NR4A3 to E‐N to T‐N (Figure 3E; see supplementary materials, Figure S2; Table S2I). Moreover, the axonal factors that were significantly modulated in the two EMC variants showed a coherent modulation trend also in T‐N versus E‐N cells (Figure 3F). These data were confirmed by RT‐qPCR assays on independent biological replicates of tBJ/ER expressing NR4A3, E‐N and T‐N (with T‐N and T‐N* yielding similar results) (see supplementary material, Figure S2).

### NR4A3 chimeras differentially bind the *SEMA3C* promoter

**Figure 4:**
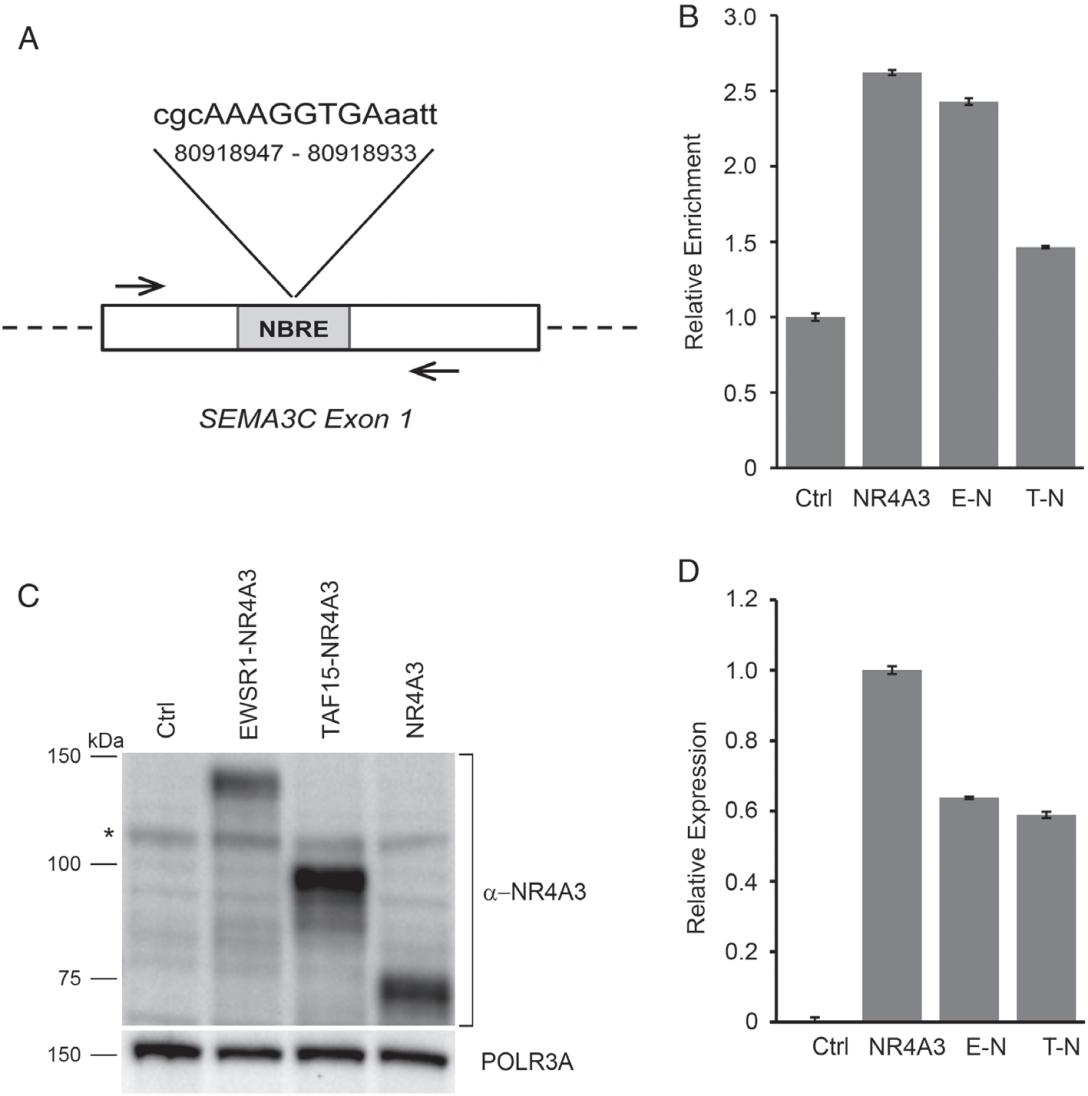
NR4A3 chimeras differentially bind the SEMA3C promoter and display diverse protein expression levels. (A) Schematic representation of the putative NR4A3 binding site identified by MatInspector on the human *SEMA3C* regulatory region. The NBRE‐like sequence and genomic coordinates are shown (human genome assembly GRCh38/hg38; chromosome 7). *A*rrows indicate the primers used in ChAP‐qPCR assays. (B) ChAP‐qPCR results of the binding of NR4A3 and NR4A3 chimeras to the indicated *SEMA3C* regulatory region. Relative enrichment for the *SEMA3C* NBRE target region in tBJ/ER cells expressing Strep‐tagged NR4A3, E‐N or T‐N versus negative control (empty vector, Ctrl) is shown. Relative enrichment indicates the amount of *SEMA3C*‐specific precipitated DNA normalized to the total input chromatin, with Ctrl set to 1. (C) Representative immunoblot for NR4A3 in tBJ/ER cells engineered to express EWSR1‐NR4A3, TAF15‐NR4A3, NR4A3, or control empty vector (Ctrl). The blot was hybridized with an anti‐N‐terminus MoAb (clone H7833). *Nonspecific band. (D) Relative expression levels of NR4A3‐related mRNAs (±SE) assessed by RT‐qPCR of the cells shown in (C).

An *in silico* analysis (MatInspector) identified several potential NR4A3 recognition sites on the regulatory regions of *SEMA* genes. In particular, *SEMA3C*, which is differentially expressed in both EMC and cell models, turned out to harbor a consensus sequence targeted by NR4A3 to regulate *CCND1* (CyclinD1) (Figure 4A) 34. To explore the possibility of a direct transcriptional control of NR4A3 over this gene, we performed ChAP assays coupled with target‐specific amplification (ChAP‐qPCR) on tBJ/ER cells engineered to express Strep‐tagged NR4A3, EWSR1‐NR4A3 or TAF15‐NR4A3. ChAP‐qPCR experiments confirmed the ability of NR4A3 to bind the predicted target on *SEMA3C*. More interestingly, the ability of NR4A3 to recognize the *SEMA3C* target region was retained by the EWSR1‐NR4A3 chimera but was impaired by TAF15‐NR4A3 (Figure 4B), in line with transcriptional profiling data.

### NR4A3 chimeric proteins are expressed at different extents

Intriguingly, in generating multiple biological replicates of the cell models, we realized that the EWSR1‐NR4A3 chimeric protein tended to be expressed at lower levels than TAF15‐NR4A3 despite essentially comparable mRNA levels (Figures 4C, D and S3). This discrepancy in RNA/protein levels was not attributable to a physiological experimental variability, as it was observed in multiple independent biological replicates with different vectors and cellular backgrounds, and it was independent of the type of antibody used (anti‐N‐terminus or anti‐C‐terminus NR4A3; anti‐Strep). EWSR1‐NR4A3 protein appeared to underexpressed also when compared to NR4A3, but in this case the difference was at least in part correlated to mRNA levels (Figure 4C,D and see supplementary material, Figure S3). Unfortunately, the lack of reliable antibodies validated for the detection by IHC of NR4A3 fusion proteins prevented us from assessing NR4A3 chimeric proteins in human tumors.

## Discussion

This study aimed at shedding light on the pathobiology of EMC, a sarcoma histotype of uncertain differentiation and unpredictable clinical behavior. Here we report that EMC expressing TAF15‐NR4A3 or EWSR1‐NR4A3 display a distinct transcriptional profile. More importantly, we provide evidence that this different transcriptional pattern can be mimicked *in vitro* by ectopic expression of the cognate chimera in oncogene‐transformed human fibroblasts, indicating that the type of NR4A3 fusion variant dictates the biology of EMC subtypes. Indeed, TAF15‐NR4A3 positive cell models displayed a more pronounced tumorigenic phenotype than EWSR1‐NR4A3 cells, as assessed by anchorage independent growth. This result is in line with the claimed more aggressive clinical behavior of TAF15‐NR4A3 EMC 12.

Both in tumors and cell models, ‘axon guidance’ and ‘neurogenesis’ were among the main functional categories that marked the difference between TAF15‐NR4A3 and EWSR1‐NR4A3 transcriptomes. NR4A3 has been associated with these pathways previously 35, but the mechanism through which NR4A3 impinges upon these phenomena is still poorly defined. We found that this intrinsic function is differentially tuned when NR4A3 fuses with either TAF15 or EWSR1, yielding a different biological outcome. In particular, compared to EWSR1‐translocated EMC, TAF15‐NR4A3 tumors tended to overexpress pro‐tumorigenic class 4, 5 and 6 *SEMAs* whilst most class 3 *SEMAs* appeared to be underexpressed. The same trend was confirmed in cell models engineered to express the two chimeric genes.

Originally identified as one of the critical processes related to connectivity during nervous system development, the axon guidance pathway was subsequently implicated in cancer, due to its involvement in cell proliferation, apoptosis, adhesion, migration, angiogenesis, and modulation of immune response 36, 37, 38, 39. Several constituents of this pathway have been found to be transcriptionally or structurally altered in tumors, and recent genomic studies implicate axon guidance as one of the most commonly affected pathways in cancer 40.

SEMAs represent a family of over 20 secreted (class 3 and in part class 4) and membrane‐bound (class 4, 5, 6, 7) proteins that work in concert with their cognate receptors (PLXNs) and co‐receptors (NRPs). By signaling to downstream kinases and GTPases, the SEMA/PLXN axis, together with EPH/EFN and SLIT/ROBO, modulate cytoskeletal dynamics and signal transduction. Furthermore, by impinging upon the extracellular *milieu*, they act as pleiotropic regulators of tissue homeostasis 41, 42. These axon guidance molecules are essentially bi‐functional as they can exhibit both attractive and/or repulsive activities 43. In the context of cancer, secreted class 3 SEMAs are in general considered to negatively regulate cell growth and angiogenesis 36, 44, 45. Accordingly, several class 3 SEMAs are reported to be inactivated or down‐regulated in tumors, which correlates with dismal prognosis 44. In contrast, class 4–6 SEMAs are considered oncogenic, providing pro‐survival and pro‐angiogenic signals 36, 44, 46, 47, and are often overexpressed in aggressive forms of cancer 44, 46, 48. Besides class 4–6 *SEMAs*, TAF15‐NR4A3 EMC also overexpressed other axon guidance‐related molecules associated with poor outcome, including *PLXNA1, CXCR4*, and *EPH/EFN* factors 49, 50, 51, 52.

Several semaphorins and plexins have been investigated as potential targets for drugs to treat cancer. In particular, a humanized antibody directed against SEMA4D (VX15/2503) has been generated recently and is currently in phase I/II clinical trials for the treatment of advanced refractory solid tumors. This antibody has demonstrated immune‐mediated antitumor effects in tumor bearing mice 53. Thus, blocking the activity of SEMA4D might represent a novel therapeutic strategy for TAF15‐NR4A3 positive EMC.

The SEMA switch detected in the cell models, which mirrors that observed in tumors, supports the notion that the type of fusion plays a key role in this axon guidance reprogramming. In an effort to gain insights on the mechanisms through which NR4A3 chimeric proteins differentially impact on axon guidance signaling, we explored the possibility of direct control of *SEMA* transcription. We identified an NR4A3 binding site in the regulatory region of *SEMA3C* and demonstrated that both NR4A3 and EWSR1‐NR4A3 efficiently bound this sequence whilst TAF15‐NR4A3 was impaired in this function. This result parallels *SEMA3C* expression (upregulated in EWSR1 versus TAF15 EMC and cell models) and suggests that the diverse fusion partners differentially tune the ability of NR4A3 to access its transcriptional targets.

On the other hand, mounting evidence points to extranuclear functions of NR4A proteins 16. In this regard, it has been reported recently that NR4A1 modulates the function of SEMA3E/PLXND1 complexes by directly binding PLXND1 and displacing SEMA3E 54. This result, which draws attention to the poorly characterized cytosolic activities of these orphan receptors, discloses the possibility that NR4A3 and relative chimeras may also interfere with axon guidance signaling *via* a similar mechanism.

Intriguingly, in generating independent replicates of the cell models, we observed that although the RNA levels for the two fusion genes were essentially comparable, the TAF15‐NR4A3 protein was expressed at higher levels than EWSR1‐NR4A3. These results indicate that the N‐terminus FET component, EWSR1 or TAF15, likely affects the expression of the chimeric protein at the post‐transcriptional level. Indeed, during neural differentiation a dichotomy in RNA/protein amounts has been observed for EWSR1, but not for TAF15, indicating a differential post‐transcriptional regulation 55. Based on the above data, it is possible that the observed phenotypes may be due, at least in part, to the different protein levels of the chimeras. Nevertheless, we believe that EWSR1 and TAF15 contribute to the biological properties of the cognate chimera also through specific, qualitative mechanisms. In fact, despite lower protein levels, EWSR1‐NR4A3 is more potent than NR4A3 in soft‐agar assays and in the modulation of axon guidance cues. This is in line with previous findings indicating that, compared to NR4A3, fusion with EWSR1 increases transcriptional activity and conveys different specificities to the chimera 16, 56. Moreover, although expressed at higher levels than EWSR1‐NR4A3, TAF15‐NR4A3 is defective in *SEMA3C* promoter binding.

As an interesting side note, the axon guidance axis is known to intersect signaling mediated by receptor kinases. In particular, secreted SEMA/PLXN have been reported to interact with and activate a number of tyrosine kinases, including MET and VEGFR 57. The connection of NR4A receptors to SEMA/PLXN and the crosstalk with receptor kinases is particularly interesting in the light of the therapeutic activity of sunitinib in EMC 3. Although small, the study hinted at a correlation between response and type of fusion, with TAF15‐NR4A3 EMC being unresponsive 3. Thus, activation of distinct sets of axon guidance cues by the two chimeras might result in a broader effect on receptor signaling pathways, thus affecting the response to suntinib. Noteworthy, *in vivo* administration of soluble SEMA3A extends the therapeutic window of sunitinib in a mouse tumor model by counteracting sunitinib‐induced hypoxia 58. Since EWSR1‐NR4A3 EMC overexpress class 3 SEMAs, the release of these soluble factors might contribute to their sensitivity to sunitinib.

In summary, our work highlights that the type of NR4A3 fusion protein affects tumor cell phenotype and dictates the engagement of different axon guidance cues which are expected to impact on tumor clinical behavior. Besides providing novel insights on the biology of EMC, these findings may lay down the basis for the development of improved criteria for patient stratification and disclose novel therapeutic avenues.

## Author contributions statement

RM, SP, and SS contributed to conception, design and supervision of the study. PC, CC, AG, SS, PAC, MAP, and PP contributed to acquisition of clinical–pathological data. MB, KF, MJ, DR, AA, VI, and GPD contributed to acquisition of molecular data. SB, PC, SP, SR, MS, and APDT contributed to acquisition of immunohistochemical data. MB and MP contributed to bioinformatic analyses. MB, SS, SP, and RM contributed to data interpretation and writing of the manuscript. All authors read and approved the manuscript.

**SUPPLEMENTARY MATERIAL ONLINE**

**Supplementary materials and methods**

**Supplementary figure legends**

**Figure S1**. Anchorage‐independent growth

**Figure S2**. Validation of the modulation of axon guidance cues in diverse tBJ/ER biological replicates

**Figure S3**. Protein and mRNA expression of NR4A3 chimeras in T‐N and T‐N* cell models

**Table S1**. Transcriptional profiling of EMC

**Table S2**. Transcriptional profiling of E‐N and T‐N cell models

## Supporting information


**Supplementary materials and methods**

**Supplementary figure legends**
**Figure S1**. Anchorage‐independent growth

**Figure S2**. Validation of the modulation of axon guidance cues in diverse tBJ/ER biological replicates

**Figure S3**. Protein and mRNA expression of NR4A3 chimeras in T‐N and T‐N* cell models

**Table S1**. Transcriptional profiling of EMC

**Table S2**. Transcriptional profiling of E‐N and T‐N cell models
