# Supplementary material for: NR4A3 fusion proteins trigger an axon guidance switch that marks the difference between EWSR1 and TAF15 translocated extraskeletal myxoid chondrosarcomas
Source: J Pathol. 2019 May 14;249(1):90–101. doi: 10.1002/path.5284 (PMC6766969; doi:10.1002/path.5284)
Supplement: Supplementary file 1 — Supplementary materials and methods [file PATH-249-90-s001.docx]

**NR4A3 fusion proteins trigger an axon guidance switch that marks the difference between EWSR1 and TAF15 translocated extraskeletal myxoid chondrosarcomas**

Brenca M *et al. J Pathol* DOI: 10.1002/path.5284

**Supplementary Materials and Methods**

Citation numbers refer to the main text

Immunohistochemistry and FISH analyses

Antibodies and staining conditions: Semaphorin 4D (Rabbit MoAb clone EP3569, 1:200, Abcam, Cambridge, UK); Plexin A4 (Rabbit polyclonal Ab, cat ab39350, 1:200, Abcam); Synaptophysin (Mouse MoAb clone DAK-Synap, 1:200, Agilent, Santa Clara, CA, USA); Reelin (Mouse MoAb, clone 142, 1:100, Millipore, Darmstadt, Germany); Nestin (Mouse MoAb, clone 4D11, 1:100, Origene, Rockville, MD, USA); NCAM1/CD56 (Mouse MoAb, clone 123C3.D5, 1:50, Agilent); Glial Fibrillary Acidic Protein (Mouse MoAb clone 6F2, 1:200, Agilent). The OptiView DAB Detection Kit on the Ventana BenchMark ULTRA platform (Ventana Medical Systems, Oro Valley, AZ, USA) and the Dako Autostainer Link48 were used to process samples.

FISH analyses were performed on FFPE tissue samples with a *EWSR1* dual-color, break-apart probe (VYSIS; Abbott Molecular, Des Plaines, IL, USA) and with two *NR4A3* specific BAC probes (BACPAC resources, C.H.O.R.I., Oakland, CA, USA): Spectrum Orange-labeled RP11-30L7 for the 5’ end and Spectrum Green-labeled RP11-30N20 for the 3’ end of *NR4A3*. TAF15-NR4A3 rearrangements were detected with a dual-color single-fusion FISH strategy with Spectrum Green-labeled *NR4A3* probes (RP11-30L7, RP11-30N20) and a Spectrum Orange-labeled 3’ *TAF15* probe (RP11-1094M14). Fluorescent labeling of BAC clones was performed with a nick-translation kit (Sigma-Aldrich, St Loius, MO, USA). A minimum of 50 non-overlapping nuclei were scored at 100X magnification with appropriate filters.

Gene Functional Annotation

For functional annotations of the genes differentially expressed in TAF15-NR4A3 *vs* EWSR1-NR4A3 EMC and T-N *vs* E-N cell models the following parameters were applied: abs.log2 FC >0.6 and p <0.05. In the case of DAVID and IPA analyses of T-N *vs* E-N cells, a more stringent FDR <0.05 instead of p<0.05 was used. The WebGestalt Suite was used for over-representation (ORA) and gene set enrichment analysis (GSEA). For both methods, we used geneontology_Biological_Process_noRedundant as a functional category. To run ORA-WebGestalt the following parameters were set: Minimum number of genes in the category: 3; Maximum number of genes in the category: 2000; FDR Method: BH; Significance Level: FDR<0.05. To perform GSEA-WebGestalt the following parameters were used: Minimum number of genes in the category: 3 for EMC tumors and 5 for cell models; Maximum number of genes in the category: 2000; Significance Level: Top 10; number of permutation:1000. Parameters used for GSEA-Broad Institute were: pre-ranked mode; predefined C5 gene set database; default settings for basic and advanced fields. Network integration analysis of the 170 genes identified as commonly differentially expressed in TAF15-NR4A3 *vs* EWSR1-NR4A3 EMC and T-N *vs* E-N engineered cell models was performed by using the NetworkAnalyst tool. The enrichment analysis was based on GO biological process database, using default settings.

Cells and constructs

U2-OS, MES-SA, VA-ES-BJ, HOS, HT-1080 and h-tert immortalized human primary fibroblasts primed to transformation by the ectopic expression of E1A and Ras oncogenes (tBJ/ER) were maintained as previously described [31,32]. Retroviral infections were performed as in [31,32]. The following cDNAs cloned into the retroviral PLPCX vector (Clontech, Takara Bio Inc, Kusatsu, Japan) were used to engineer tBJ/ER cells: full-length *NR4A3*; E-N, corresponding to the *EWSR1-NR4A3* fusion (exon 12 ENST00000397938.6-exon 3, ENST00000395097.6); T-N, corresponding to the *TAF15-NR4A3* fusion (exon 6 ENST00000605844.5- part of intron 2 ENST00000395097.6), which was detected in EMC cases #11 and #12; T-N*, corresponding to the commonest *TAF15-NR4A3* fusion (exon 6 ENST00000605844.5-exon 3, ENST00000395097.6). Both T-N and T-N* encode the whole coding sequence of *NR4A3* (exons 3-8); in T-N, this is preceded by a short cryptic exon located in *NR4A3* intron 2 of ENST00000395097.6 isoform, thus encoding 25 additional amino acids prior *NR4A3* ATG (GPHHLFSSQDFIPYMHDSIRFGNVD). Both untagged and Strep-tagged versions of these plasmids were used.

For transcriptional profiling 4 independent replicates for E-N and T-N and 3 replicates for NR4A3 were generated by separate viral infections of target cells.

Anchorage-independent growth assay was carried out in soft agar-semisolid medium as previously described [31]. Colonies were scored at 100X magnification 8 days after plating and size and number of colonies/field (cutoff size > 30 µm) were estimated. A minimum of 20 non-overlapping fields of 3 independent replicates were scrutinized blindly by two investigators.

Protein analysis

For western blot analysis, cells were lysed in 20mM Hepes (pH 7.9), 400 mM NaCl, 1mM EDTA, 1% NP40, 1mM DTT, supplemented with protease inhibitor cocktail (Complete Protease Inhibitor, Roche, Basel, Switzerland). Protein lysates (40 µg) were loaded on 4%–15% gradient TGX Stain-Free™ Gel (Bio-Rad, Hercules, CA, USA) and electroblotted onto 0.45 µm Nitrocellulose Membrane (Sartorius, Goettingen, Germany). Membranes were probed with the following anti-NR4A3 antibodies: Mouse MoAb clone H7833 (R&D Systems, Minneapolis, MN, USA) targeting the N-terminus of the protein; Mouse MoAb clone OTI5C2 (Origene) targeting the C-terminal portion of NR4A3. An anti-POLR3A antibody (Rabbit MoAb, clone D5Y2D, Cell Signaling) was used to normalize total protein load. A mouse MoAb anti-Strep Tag (clone GT661, AbCam) was also used to visualize Strep-tagged constructs.

Quantitative RT-PCR (RT-qPCR) and Targeted transcriptional array

Relative mRNA expression levels of *NR4A3*, related fusions and of a set of *SEMAs* was assessed in tBJ/ER cell models by RT-qPCR on a CFX96 Real-Time Apparatus (Bio-Rad).

Total RNA was reverse transcribed into cDNA using the SuperScript III Reverse Transcriptase (ThermoFisher Scientific, Waltham, MA, USA) and random examers. RT-qPCR was performed with SSO Fast EvaGreen Supermix (Bio-Rad). At least 3 independent biological replicates were analyzed. The comparative Ct (ΔΔCt) method and the geometric average of a set of three endogenous references (*SF3A1, PPIA* and *MRPL19*) were used to calculate gene relative expression. The primers used are indicated below.

NR4A3-Fw: 5’ ACTGCCCAGTAGACAAGAGACG 3’;

NR4A3-Rev: 5’ GTTTGGAAGGCAGACGACCTCT 3’;

SEMA3A-Fw: 5’ GGTGCCTTATCAAGGAAGAGTCC 3’;

SEMA3A-Rev: 5’ TACATGGCTGGATGACTTCTTGC 3’.

SEMA3C-Fw: 5’ ACCCACTGACTCAATGCAGAGG 3’;

SEMA3C-Rev: 5’ CAGCCACTTGATAGATGCCTGC 3’.

SEMA4G-Fw: 5’ CTGGATGCTGAAACCTCAAGCC 3’;

SEMA4G-Rev: 5’ GCAAAGACAGCCTGGATCTCTG 3’;

SEMA5A-Fw: 5’ CCTGAAGAGGTGCCAGTTCTAC 3’;

SEMA5A-Rev: 5’ GCAGAGATGCTCTGTTCCCACT 3’;

SEMA6A-Fw: 5’ ACCTGTATTGCCTCCAGAGACC 3’;

SEMA6A-Rev: 5’ CCAGACCATCTGTATTGCCACG 3’.

SF3A1-Fw: 5’ ACCTTCTAAGCCAGTTGTGGG 3’;

SF3A1-Rev: 5’ TAGCTTCAAATTCAGGCCCGT 3’.

PPIA-Fw: 5’ TCTGCACTGCCAAGACTGAG 3’;

PPIA-Rev: 5’ TGGTCTTGCCATTCCTGGAC 3’.

MRPL19-Fw: 5’ CAGGAAGAGGACTTGGAGTAC 3’;

MRPL19-Rev: 5’ GCTATCATCCAGCCGTTTCTCTA 3’.

Targeted transcriptional array analysis was performed on a subset of EMC for which suitable material was available (3 EWSR1 and 3 TAF15). Total RNA (200 ng) was retro-transcribed and amplified using the Sigma TransPlex Whole Transcriptome Amplification Kit. cDNA labeling was performed with Agilent SureTag DNA labeling Kit. cDNA libraries were hybridized to Agilent SurePrint G3 Human Gene Expression 8x60K Microarrays. The Agilent Image Analysis Software was used to extract raw data and Biomedical Genomics Workbench was used for data analysis.

Chromatin Affinity Purification-quantitative PCR (ChAP-qPCR)

Chromatin Affinity Purification was performed on tBJ/ER cell models engineered by retroviral infection to ectopically express Strep-tagged NR4A3, EWSR1-NR4A3 or TAF15-NR4A3. Three-10 cm plates at 80% confluence were used per ChAP-qPCR. Chromatin cross-linking was performed as previously described [59] with minor modifications. Formaldehyde was added directly to cell culture media at a final concentration of 1% for 5 min at room temperature (RT). Cross-linking reaction was stopped by cold PBS rinsing and addition of 0.125M glycine (5 min at RT). After additional rinsing, cells were scraped in PBS supplemented with protease inhibitor cocktail (Complete Protease Inhibitor, Roche), pooled and collected by centrifugation at 4 °C (10 min at 1000 rpm). Cells were then swelled in 1 ml lysis buffer [5 mM Pipes pH 8.0, 85 mM KCl, 0.5% NP40] supplemented with protease inhibitors (Complete, Roche) and incubated on for 3 min at 4°C. Nuclei were collected by microcentrifugation (5 min at 3000 rpm), resuspended in 1 ml sonication buffer [1% SDS, 10 mM EDTA, 50 mM Tris⋅HCl pH 8.1] and incubated for 30 min, rocking in cold room. Chromatin was sheared with a Covaris M220 Focused-ultrasonicator (Covaris Inc., Woburn MA, USA) for 30 min to a fragment size of 100–1000 bp (Peak Power: 50 W; Duty factor: 20%; Cycles/burst: 200; Temperature: 8°C). Sheared DNA (30 µg in 500 µl) was pre-cleared with 50 µl of SP sepharose® Fast Flow (GE Healthcare, Chicago, IL, USA) (2 h at 4°C). Pre-cleared chromatin (450 µl) was precipitated by addition of 50 µl of 50% slurry Strep-Tactin sepharose (IBA Lifesciences, Goettingen, Germany) (1 h at 4°C). Fifty µl of pre-cleared chromatin were saved as total input and processed with the eluted sample beginning at the crosslink reversal step. Complexes were washed 5 times in wash buffer 1 [150 mM NaCl, 2 mM EDTA, 20 mM Tris⋅HCl pH 8.1, 0.1% SDS, 1% TritonX100] , 4 times in wash buffer 2 [500 mM NaCl, 2 mM EDTA, 20 mM Tris⋅HCl pH 8.1, 0.1% SDS, 1% TritonX100] , once in TE [10 mM Tris HCl pH 8.1; 1 mM EDTA]. Finally, samples were eluted in 400 µl Elution Buffer [1% SDS, 100 mM NaHCO3] (1 h rocking at RT). Cross-links were reversed by addition of NaCl to a final concentration of 200 mM (16 h at 65°C). RNA and proteins were removed by addition of RNase A (20 μg/sample) and proteinase K (100 μg/sample) (2 h at 45°C). Samples were extracted with phenol:chloroform:isoamyl alcohol (25:24:1) and then precipitated with 1/10th vol of 3 M sodium acetate (pH 5.3) and 2 vol of ethanol in the presence of glycogen (15 μg/sample). Pellets were collected by microcentrifugation, resuspended in 40 μl of H2O, and analyzed by qPCR. Total input samples were resuspended in 40 μl of H2O and then diluted 1:30 before PCR.

Quantification of the precipitated DNA was performed by EvaGreen-based real-time qPCR with the following primers:

SEMA3C(NBRE)-Fw: 5’ ATGACCAATTTAGCTTACCGAGG 3’;

SEMA3C(NBRE)-Rev: 5’ TGCAGCGCTGAGATTCCTTT 3’.

GAPDH-Fw: 5’ TACTAGCGGTTTTACGGGC 3’;

GAPDH-Rev: 5’TCGAACAGGAGGAGCAGAGAGCGA 3’.

The fraction of the target DNA recovered from the input was measured by comparing the threshold cycle (CT) of the precipitated sample to a dilution of its own input, and was expressed as “Relative Enrichment”. Relative Enrichment indicates the amount of *SEMA3C*-specific precipitated DNA normalized to the total input chromatin, with negative control (empty vector) used as a reference. The level of background was estimated by PCR amplification of an unrelated genomic region (*GAPDH* exon 1). ChAP reactions were considered informative when no selective amplification was observed for the unrelated *GAPDH* genomic region.
