# Supplementary figures and images for: NR4A3 fusion proteins trigger an axon guidance switch that marks the difference between EWSR1 and TAF15 translocated extraskeletal myxoid chondrosarcomas
Source: J Pathol. 2019 May 14;249(1):90–101. doi: 10.1002/path.5284 (PMC6766969; doi:10.1002/path.5284)

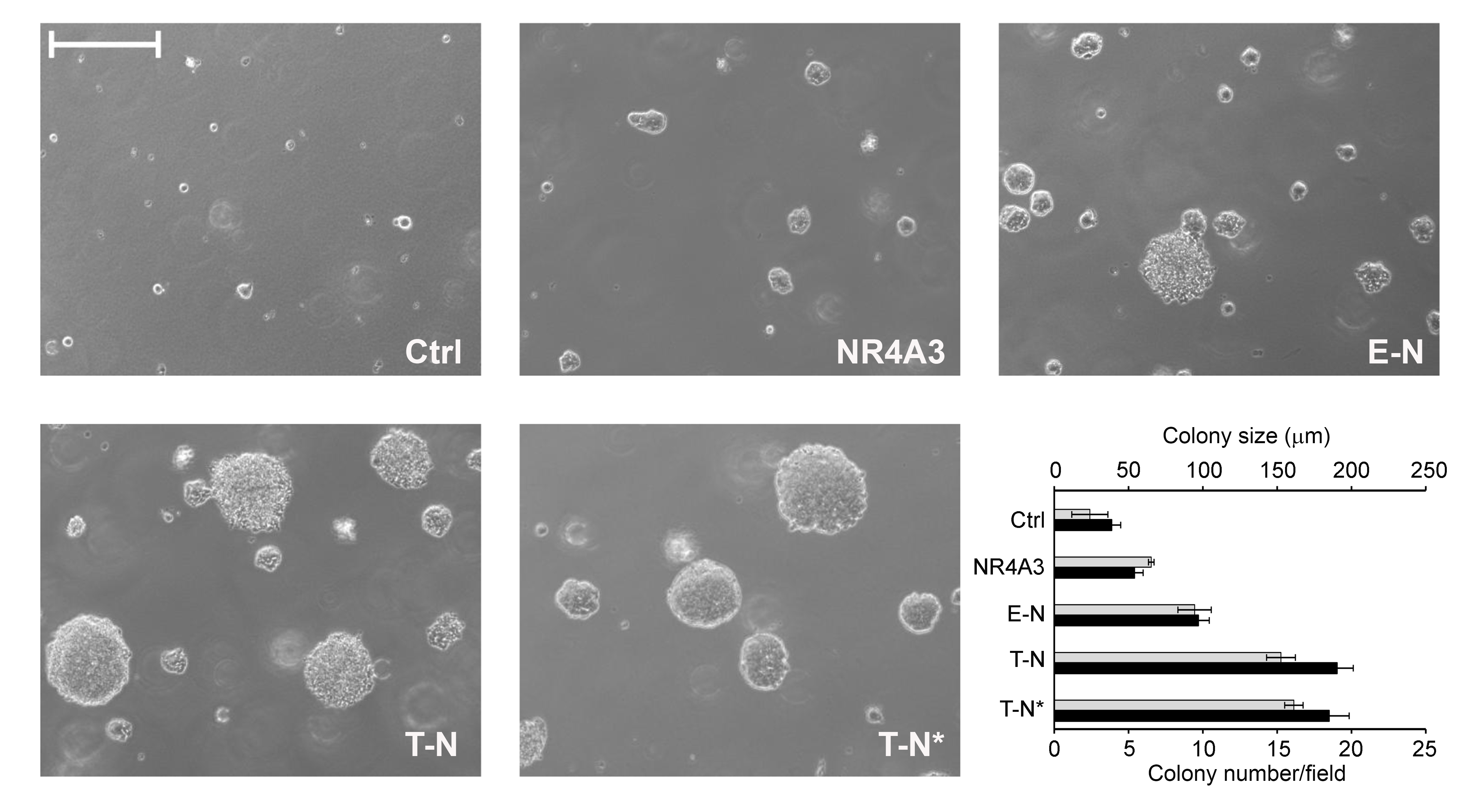

Supplement: Supplementary file 2 — Supplementary figure legends Figure S1. Anchorage‐independent growth [file PATH-249-90-s002.tif]

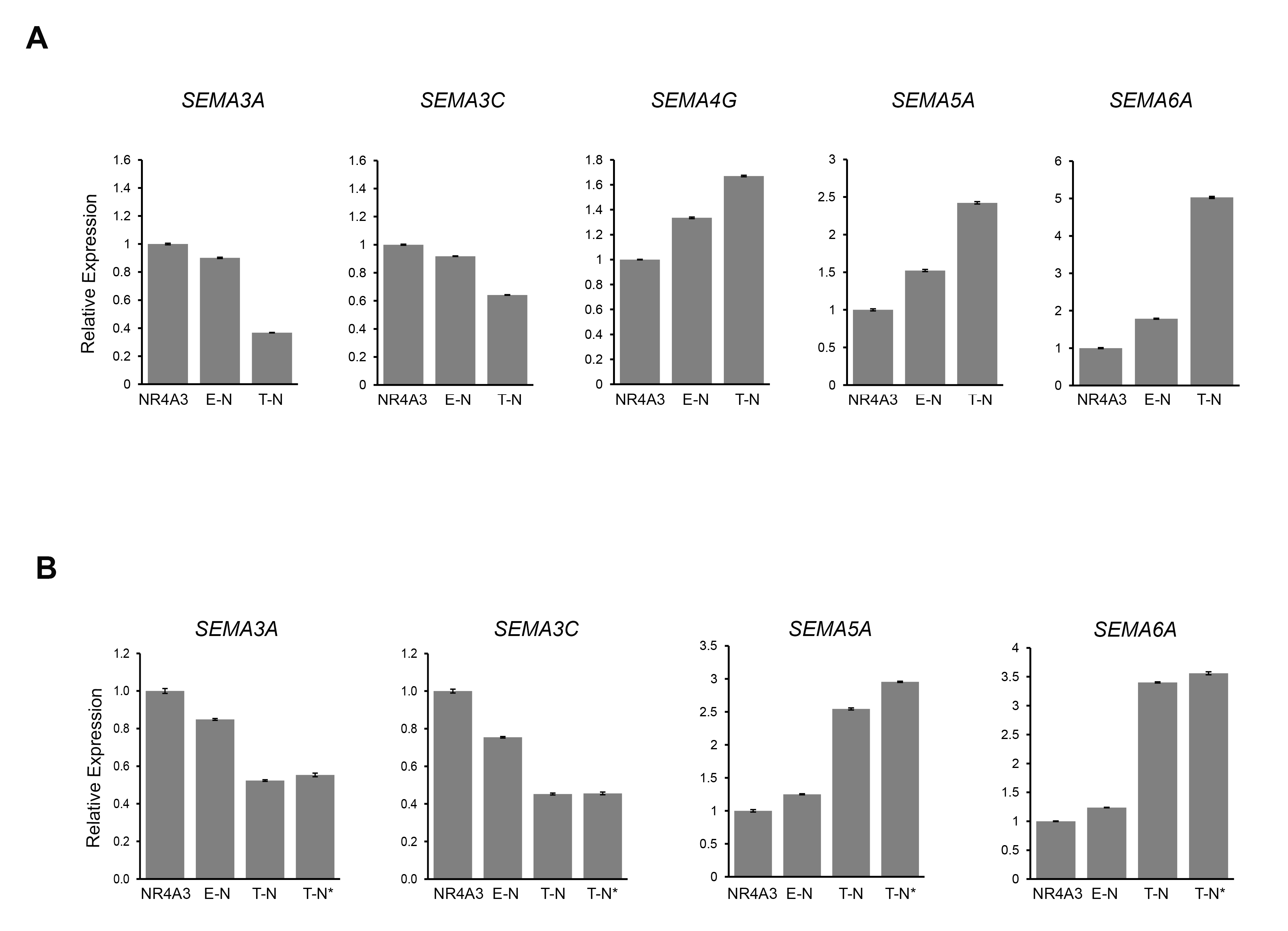

Supplement: Supplementary file 3 — Figure S2. Validation of the modulation of axon guidance cues in diverse tBJ/ER biological replicates [file PATH-249-90-s003.tif]

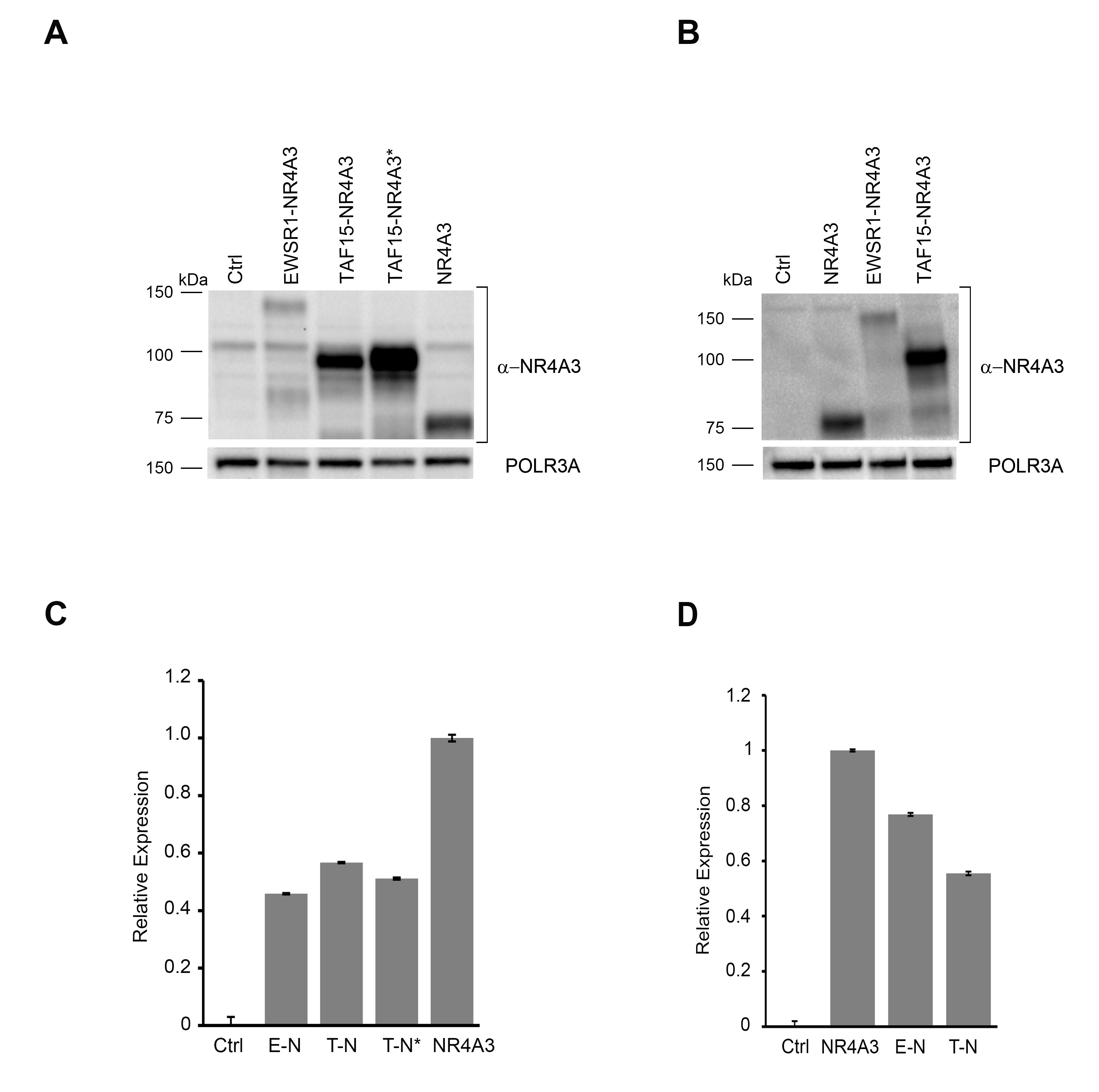

Supplement: Supplementary file 4 — Figure S3. Protein and mRNA expression of NR4A3 chimeras in T‐N and T‐N* cell models [file PATH-249-90-s004.tif]
